# Microtubular Assessment of C6 Rat Glioma Cell Spheroids Developed in Transparent Liquid Marbles or Hanging Drops

**DOI:** 10.3390/biology11040492

**Published:** 2022-03-23

**Authors:** Arianna Langella, Sergio Domenico Gadau, Elisa Serra, Daniela Bebbere, Sergio Ledda

**Affiliations:** Department of Veterinary Medicine, University of Sassari, Via Vienna 2, 07100 Sassari, Italy; arilangi@hotmail.it (A.L.); eliserra@uniss.it (E.S.); dbebbere@uniss.it (D.B.); giodi@uniss.it (S.L.)

**Keywords:** liquid marble, hanging drops, glioma cells, 3D culture, microtubular assessment

## Abstract

**Simple Summary:**

Due to its high refractoriness to therapies, glioblastoma brain tumour is frequently used as a model to develop new therapeutic approaches. Many of these treatments may target the microtubular network of the cell, also considering that tubulin post-translational modifications (PTMs) are markers of tumour plasticity. The two-dimensional (2D) culture systems are now being replaced by three-dimensional (3D) systems capable of mimicking in vivo conditions. In this work, spheroids were developed from C6 rat glioma cells (RGCs) using two 3D systems: liquid marbles (LMs) or hanging drops (HD) and analysed in terms of the morphology and behaviour of the two main tubulin PTMs, tyrosinated α-tubulin (Tyr-T) and acetylated α-tubulin (Ac-T). RGCs spontaneously formed spheroids more rapidly in the LM than in the HD system. An increase in Tyr-T and Ac-T was observed in both the HD and LM system during IVC, with the highest values shown in LM spheroids. In conclusion, the present work shows that the LM 3D system boosts the induction and maintenance of a high plasticity state in glioma cells and could provide a novel approach to set up a biological system to evaluate new anticancer therapies and advance knowledge on glioblastoma.

**Abstract:**

Glioblastoma is a brain tumour frequently used as an experimental model to exploit innovative therapeutic approaches due to its high lethality and refractoriness to therapies. Part of these innovative anticancer therapies address cytoskeletal microtubules (MTs) since specific tubulin post-translational modifications (PTMs) are considered markers of tumour plasticity. In vitro studies, which traditionally employ two-dimensional (2D) culture systems, are now being replaced by three-dimensional (3D) systems that more closely mimic in vivo physiological conditions and allow a better understanding of the signalling between cells. In this work, we compared 2 liquid base 3D methods for the generation of spheroids from C6 rat glioma cells (RGCs) using 30 µL of liquid marble (LM) or the hanging drops (HDs), which contained 2 different cell numbers (5000 or 15,000). After 24 or 48 h of in vitro culture (IVC), the morphology of the spheroids was observed and the behaviour of the two main tubulin PTMs, tyrosinated α-tubulin (Tyr-T) and acetylated α-tubulin (Ac-T), was evaluated by fluorescence and Western blot (WB). RGCs spontaneously formed spherical agglomerates more rapidly in the LM than in the HD system. Cell density influenced the size of the spheroids, which reached a larger size (> of 300 µm Ø), with 15,000 cells compared to 5000 cells (150 µm Ø). Moreover, an increase in Tyr-T and Ac-T was observed in both the HD and LM system from 24 to 48 h, with the highest values shown in the 48 h/LM spheroids of 5000 cells (*p* < 0.05). In conclusion, by comparing the morphology and microtubular architecture of spheroids from C6 rat glioma cells developed by LM or HD methodology, our findings demonstrate that the use of a fumed silica microbioreactor boosts the induction and maintenance of a high plasticity state in glioma cells. RGCs cultured in LM express levels of tubulin PTMs that can be used to evaluate the efficacy of new anticancer therapies.

## 1. Introduction

Glioblastoma multiforme (GBM) is the most common and aggressive malignant primary brain tumour in humans, characterized by the highest lethality and the lowest life expectancy [1]. One peculiarity of GBM, which often underlies the ineffectiveness of therapeutic treatments, is the ability of cancer cells to invade surrounding brain tissues, thus emphasizing the key role of the interaction between cellular elements and the extracellular matrix [2,3]. In the last decades, studies of different types of tumours, including GBM, have mainly been performed with 2D cell culture systems, given the simple establishment of culture conditions, the high reproducibility, and the low cost [4,5]. However, 2D cell cultures show some limitations due to their inability to reproduce some tumour characteristics, such as the three-dimensionality and interaction with the extracellular matrix. Starting from the assumption that cells are organized within tissues in a 3D structure that affects both cell–cell and cell–extracellular matrix interactions, we have recently seen a significant increase in in vitro 3D cellular models [6,7]. In this sense, replicating the complex structural organization of the brain in vitro, moving from traditional 2D to 3D models, would help to better understand the invasion mechanisms of GBM cells [8,9]. Further, 3D systems can be scaffold-based, in which cells grow on an artificial 3D structure, or scaffold-free, when cells produce their own extracellular matrix and grow in a 3D manner [10]. Among the scaffold-free 3D systems, spheroids represent one of the best studied 3D models in recent years. Spheroids are made of cellular aggregates that can grow in suspension or be included in a 3D matrix [11]. Many types of mammalian cells can aggregate and differentiate into 3D multicellular spheroids when cultured in suspension or in a non-adhesive environment. Compared to conventional monolayer cultures, multicellular spheroids better resemble real tissues in terms of their structural and functional properties. Many progenitor cells show significantly enhanced viability and functional performance when grown as spheroids [12]. Multicellular spheroids are ideal building units for tissue reconstruction or to better understand tumour evolution in vitro. Current methods to produce spheroids are the results of studies that started in 1906 with the hanging drops (HDs) tissue culture approach described by Ross Harrison (1906) in an attempt to recapitulate organogenesis in culture. Among the different systems, four major liquid-based methods are used to culture cancer cell spheroids: (i) the rotary wall vessel and spinner flask systems use rotary devices to constantly keep cells in suspension for aggregation into spheroids with random sizes; (ii) the hanging drop array methods use gravitational-mediated aggregation of cells in the apex region of drops hanging from a plate; (iii) cells are dropped in a non-adhesive well and spontaneously aggregate to form a compacted spheroid with a well-defined size; and (iv) microfluidic devices where the cells are placed in microchannels with a free perfusion system, allowing the distribution of oxygen and nutrients, and the elimination of metabolic waste [13]. Regular bacteriological-type Petri dishes and certain ELISA 96-well plates made of non-adhesive plastics are suitable for the generation of spheroids [14]. Comparisons between liquid-based 3D culture methods for the generation of spheroids, showing their limitations and advantages, have previously been reported in detail [15,16,17].

These experimental 3D models have seen widespread use in recent years for the screening of new therapeutic approaches. This aspect is fundamental considering glioblastoma, which is endowed with high refractoriness to chemotherapy treatments [18]. It is well known that many anticancer drugs target the cellular cytoskeleton and the microtubular network [19,20,21,22]. The microtubular network in glioblastoma cells sparks interest both from the point of view of experimental therapeutic approaches and from the point of view of tumour cell biology [11,23]. Recently, we carried out studies on 2D models on the content, distribution, and interaction with motor proteins of the most widespread PTM tubulins in rat glioblastoma cells [24,25]. In these papers, it was shown that tyrosinated and acetylated tubulin were among the most abundant PTM tubulins in C6 cells. In the past years, some 3D models have already been tested in the study of glioblastoma [26,27,28,29]. Most of these studies were conducted on the brain biopsies of patients with glioblastoma, from which, using Mem-agar or Matrigel, spheroids were obtained [28,30,31,32]. Here, for the first time, we propose a protocol to obtain spheroids of C6 RGCs by encapsulation in super-hydrophobic microbioreactors. The microbioreactors, defined as liquid marbles (LMs), are formed by enveloping cells suspended in a drop of medium with hydrophobic-treated fumed silica powder, with particle size of 1 μm, to form an elastic shell with fine pores [33]. Fumed silica particles adhere to the surface of the medium drop, isolating the liquid core from the supporting surface while allowing optimal gas exchange between the interior liquid and the surrounding environment. The coating material acts as a confined space, which is non-adhesive and allows cells to freely interact with each other [34]. Such a protocol has previously been shown to support the growth of living microorganisms, tumour spheroids, fibroblasts, red blood cells, embryonic stem cells, and oocytes [34,35,36,37,38]. The advantages of the proposed system include the transparent nature of the LM, which allows for easy visual assessment of the cells; low cost; self-repairing ability of the LM coating; low cell culture medium consumption; high reproducibility; and gas permeability. The present study compared the morphology and microtubular architecture of spheroids from C6 rat glioma cells developed by the LM or HD methodology. Considering that some tubulin post-translational modifications (PTMs) are predominant in glioblastoma cells [36], we characterized the behaviour of tyrosinated α-tubulin (Tyr-T) and acetylated α-tubulin (Ac-T) in HD and LM RGC spheroids.

## 2. Materials and Methods

All reagents used in this study were purchased from commercial sources. In detail, primary antibodies and secondary AP-conjugated antibodies were purchased from Sigma Aldrich and Millipore, now Merck Millipore (Merck KGaA, Darmstadt, Germany). The Alexa Fluor secondary antibodies were purchased from Thermo Fisher Scientific (Thermo Fisher, Waltham, MA, USA). The commercial origin of the other chemicals is specified in the text.

### 2.1. Cell Culture

Undifferentiated C6 RGCs (American Type Culture Collection, Rockville, MD, USA) were initially cultured in monolayers as previously indicated [25,39,40]. Briefly, cells were grown in phenol-red-free RPMI-1640 medium supplemented with 10% heat-inactivated foetal calf serum, 2 mM L-glutamine, 100 U/mL penicillin, and 100 µg/mL streptomycin on culture plates and placed in a 95% air–5% CO_2_ humidified incubator at 37 °C. The 2D culture of RGCs was then used to make 3D cell cultures for subsequent analyses.

### 2.2. 3D Cell Culture

Cells were grown with two different 3D techniques, hanging drops (HDs) or liquid marble (LM), to generate glioma tumour spheroids. Once cells reached a 70–80% confluence in the 2D system, they were detached with 1 mL of 5 mM PBS/EDTA and transferred to a 15 mL Falcon tube. A 20 μL aliquot of the suspension was taken and added to 20 μL of Trypan Blue to identify the dead cells. Cells were counted with a counting chamber (Burker’s chamber) under a light microscope. To obtain the experimental cell number (5000 and 15,000), they were centrifuged at 1000 rpm for 4 min, the supernatant was removed by inversion, and the cells were resuspended in fresh medium to make up the volume. The number of cells (5000 and 15,000) were selected on the bases of previous experiments in our laboratory. For both the HD and LM techniques, drops with a volume of 30 μL were used containing the final number of 5000 (dilution 1:3) and 15,000 cells. Therefore, groups with different cell densities, lengths of IVC, and 3D systems were created.

#### 2.2.1. Hanging Drops (HDs)

To set up suspended drops, non-adherent Petri dishes with a diameter of 30 mm were used. The bottom of each plate was used as a hydration chamber to reduce the phenomenon of evaporation by placing 2 mL of PBS inside. The lid of the Petri dish was turned upside down and 5 drops of 30 μL each were placed on its bottom, sufficiently spaced from each other, and containing 5000 or 15,000 cells. The plate lid was then inverted and placed on the lower chamber filled with PBS. In this way, a “pending drop” was formed, and the aggregation of cells was induced because they were pushed towards the bottom of the drop by gravity. The plates were then placed for 24 or 48 h in a 95% air/5% CO_2_ humidified incubator.

#### 2.2.2. Liquid Marble (LM)

The formation of LM requires the use of super-hydrophobic powders. For our studies we used a powder consisting of treated fumed silica (CAB-O-SIL TS-530, Cabot, Italy), characterized by a superhydrophobic surface. The powder adheres to the cell suspension and thus constitutes a low moisture coating. A CAB-O-SIL TS-530 powder bed, with a particle size of 1 μm, was set up in a Petri dish. A single drop of 30 μL, containing 5000 or 15,000 cells, was dispensed on the powder bed. The plate was gently rotated in a circular motion (rolling) to ensure that the dust particles completely covered the surface of the liquid drop and formed a “liquid marble”. The marbles were collected using a 1000 μL tip, cut on the edge, to adapt it to the diameter of the marble. Each LM was placed inside a non-adhering 30 mm^2^ Petri dish and 4 of these, containing a single marble, were placed inside a 90 mm^2^ Petri dish, which was used as a hydration chamber, to reduce the phenomenon of evaporation. PBS was placed inside to cover the entire surface. The plates containing LM were then incubated at 37 °C/5% CO_2_/95% humidity for 24 or 48 h, respectively.

The spheroids obtained with the 2 3D systems (HD or LM) were manually evaluated for their degree of clustering and an estimation of the diameters using the ImageJ freeware (NIH, Bethesda, MD, USA) and then collected for subsequent immunocytochemistry and Western blot analysis. The diameter of 20 spheroids was measured for each experimental group; the number of samples was selected on the basis of previous works [15,41,42].

### 2.3. Immunofluorescence and Confocal Microscope Acquisition

The HD and LM spheroids grown in vitro were fixed in a solution containing 2% paraformaldehyde and 0.1%, Triton-X-100 for 20 min at 4 °C. Immunofluorescence was performed as previously described [25,43,44]. Briefly, to highlight the main tubulin PTMs, the following primary antibodies were used: anti-total α-tubulin (dil.1:800, catalog # T6199, clone DM1A); monoclonal anti-tyrosine α-tubulin (1:800, catalog # T9028; clone, TUB-1A2) anti-acetylated α-tubulin (1:800; catalog # T7451; clone 6-11B-1), with overnight incubation. After incubation, the spheroids were washed in PBS/2% FCS for 15 min. As secondary antibodies, anti-mouse FITC/TRITC-AlexaFluor 488/594 were used. Subsequently, the spheroids were washed and mounted on a slide in a medium containing 50% glycerol, 2.5 mg/mL sodium azide, and 1 mg/mL blue from Hoechst 33342, using Vaseline bearings to prevent the compression of the samples. Images were obtained with a confocal laser scanning microscope from Leica (TCS SP5 DMI 6000CS, Leica Microsystems GmbH, Wetzlar, Germany) using a 40/60× oil objective. FITC was excited at 488 nm and emission was detected between 510 and 550 nm. TRITC was excited at 568 nm and emission was detected between 585 and 640 nm. Images of 1024 × 1024-pixel resolution, 8 bits depth, were acquired. All images were acquired with constant settings of the confocal microscope (Pinhole [airy] 1.00; Frame-Average 1; Line-Average 4; Laser Line UV (405) 21.00%; Laser Line Visible (543) 40.00%; Laser Line Visible (488) 32.00%). Five replicates from all the groups of HD and LM were analysed.

### 2.4. Western Blot (WB)

WB was performed as previously described [40,43]. Briefly, we collected spheroids from about 40/50 HD and from LM, after 24 or 48 h of IVC. The spheroids were then placed in lysis buffer (5 mM TRIS HCl, 2 mM EGTA, 0.1 mM phenyl-methyl-sulfonyl fluoride, pH 8.0) and a protease inhibitor was added. For each sample, the total protein concentration was determined, and 100 μg protein aliquots for each lane were loaded and separated in 10% SDS-PAGE gels, and then transferred to nitrocellulose membranes. These were then incubated overnight at 4 °C with the following primary antibodies: (1) total anti-α-tubulin (monoclonal, clone DM1A, 1:500); (2) anti α-tubulin tyrosine (monoclonal, clone TUB-1A2, 1:500); and (3) anti-acetylated α-tubulin (monoclonal, clone 6-11B-1, 1:500). After a few washes, the membranes were incubated for 1 h at 37 °C with the secondary anti-mouse antibodies conjugated with alkaline phosphatase. Blots were detected with a chromogen (NBT/BCIP). The optical density of the bands was evaluated using the ImageJ software, on three blots for each tubulin (total alpha, tyrosinated, and acetylated). The optical density values of the blot bands obtained for the total alpha, tyrosinated, and acetylated tubulins were normalized with respect to the optical density of the β-actin loading control bands.

### 2.5. Statistical Analysis

After analysis of the homogeneity of variance by Levene’s test, data were analysed by the ANOVA general linear model followed by Tukey’s post hoc comparison with the statistical software program Statgraphic Centurion XV (version 15.2.06 for Windows; StatPoint, Inc., Herndon, VA, USA). A probability of *p* ≤ 0.05 was considered the minimum level of significance. All results are expressed as mean ± SD.

## 3. Results

### 3.1. Morphology Features of Spheroids

**Figure 1:**
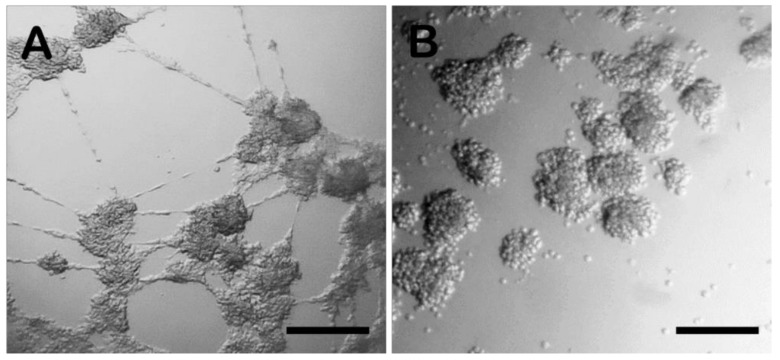
HD and LM spheroids. After 24 h of IVC, evident cytoplasmic prolongations (black arrows) between the spheroids were observed in HD containing 15,000 cells (**A**) in comparison with LM 15,000 cell spheroids (**B**) where the cytoplasmic processes disappeared. Bar = 100 µm. Images were manually analysed by Image J and are representative of replicates (*n* = 5) of each group.

**Figure 2:**
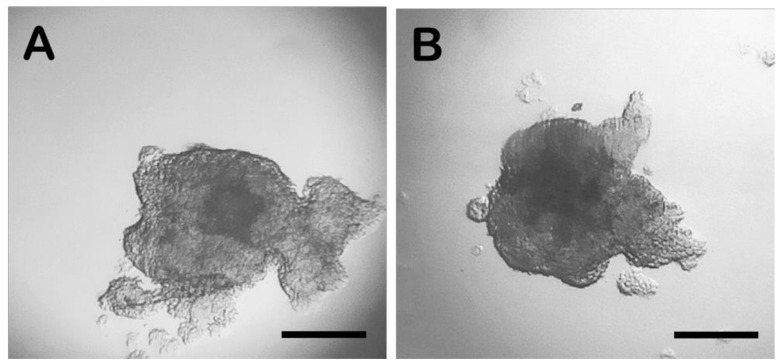
Microscopic observation of the spheroids after 48 h of IVC from drops containing 15,000 cells. (**A**) HD spheroid; (**B**) LM spheroid. Bar = 100 µm. Images were manually analysed by Jmage J and are representative of replicates (*n* = 5) of each group.

The analysis of spheroid formation allowed an evaluation of the features of the two microbioreactors by varying the concentration of the cells and the length of IVC. LM displayed more rapid cell aggregation after 24 h of incubation compared to the HD technique (Figure 1). In the HD spheroids, evident cytoplasmic prolongations between the spheroids were observed (Figure 1A), both in drops containing 5000 and 15,000 cells, while these cytoplasmic intercellular extensions were completely absent in the LM drop system (Figure 1B). After 24 h, in the HD culture system, the compactness of the cells was lower than at 48 h, when more compact aggregates were observed. However, at 48 h, similar cell clustering was observed, and the spheroid morphology was equivalent in the 2 systems (Figure 2). During the early stages of culture, cells assumed and maintained a spherical conformation, showing a coherent original phenotype, which instead tended to change during the prolonged culture, with a flattening of the peripheral cells (Figure 1 and Figure 2).

**Figure 3:**
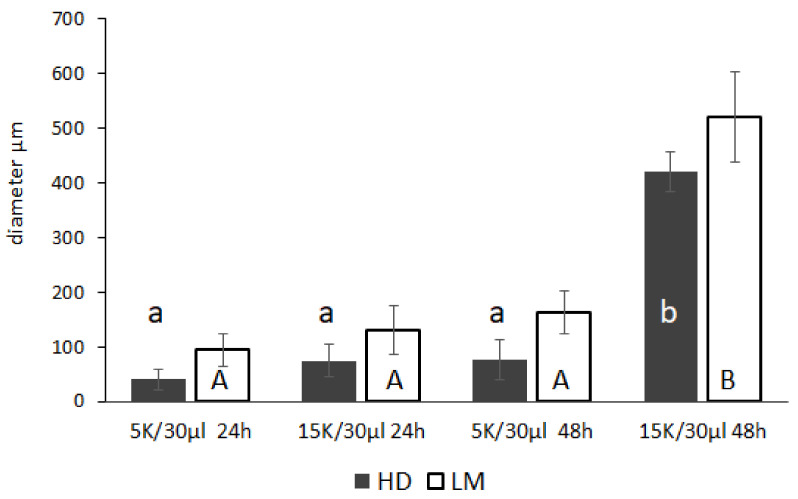
Clustering during IVC of C6 RGCs using the HD or LM techniques: evaluation of the diameter of spheroids at 24 and 48 h of IVC. 5 K = 5000 cells; 15 K = 15,000 cells. HD = hanging drops; LM = liquid marble. The data represent the mean ± standard deviation (SD) (*n* = 20). Significant differences among groups were assessed by the ANOVA general linear model followed by Tukey’s post hoc comparisons. Different letters indicate a significant difference (*p* < 0.05) between groups; lowercase letters refer to differences within the HD group, uppercase letters to the LM group. A vs. B = *p* < 0.05; a vs. b = *p* < 0.05.

As shown in Figure 3, the diameters of the spheroids depended on the cell number and length of incubation, with the spheroids obtained with 15,000 cells reaching 400 and 500 µm in HD and LM, respectively, after 48 h of IVC. The number of spheroids per drop also changed during the time of incubation in both the HD and LM systems: 15–20 spheroids formed after 24 h of culture, while 3–6 spheroids were observed after 48 h, due to the growth and aggregation of smaller spheroids. The coefficients of variation of all experimental groups are reported in Supplementary Materials Table S1.

### 3.2. Immunofluorescence Staining

**Figure 4:**
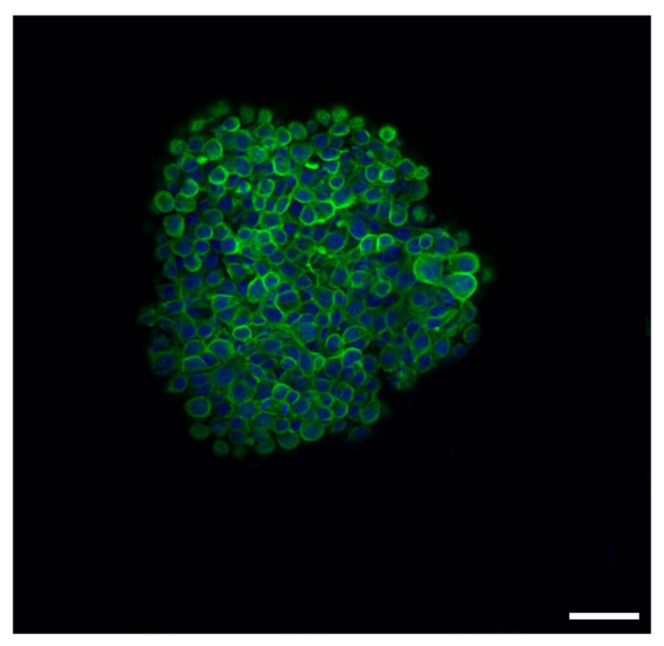
Fluorescence with total anti α-tubulin. The 3D shape and the cellular aggregate forming the spheroid are visible under confocal microscope examination. Bar = 50 μm.

A qualitative analysis was carried out using immunofluorescence. Using the anti-total α-tubulin antibody, the three-dimensional aspect of the spheroid was highlighted, with a defined cell agglomerate and good fluorescence immunoreactivity, especially in the peripheral part of the spheroid, while in the inner part, the fluorescence intensity was less marked (Figure 4).

**Figure 5:**
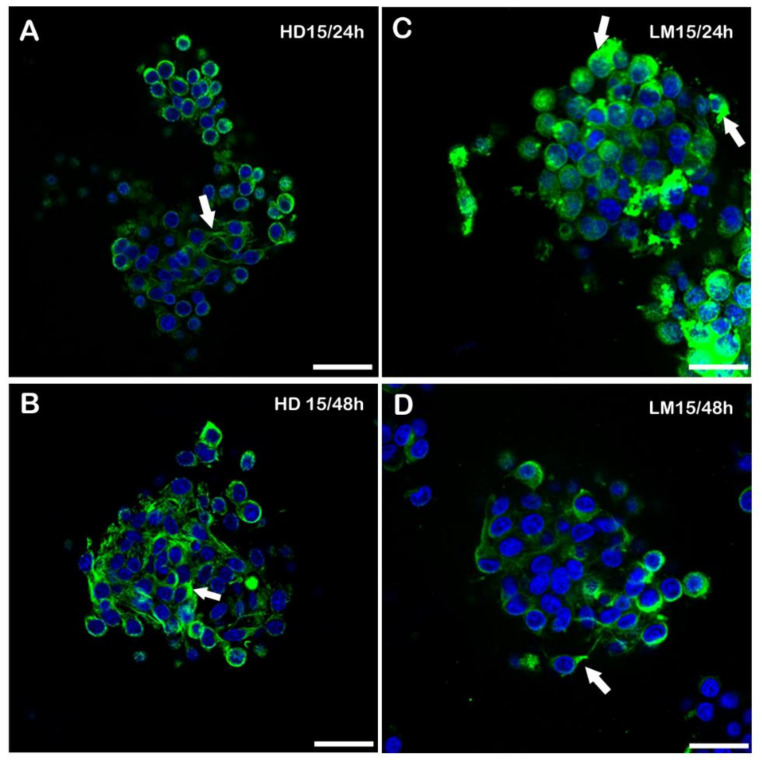
Immunofluorescence for Tyr-T in the 15,000 cell concentration spheroids. In the left column, HD spheroids at 15000/24–48 h (**A**,**B**). In the right column, confocal pictures of LM spheroids at 15,000/24–48 h (**C**,**D**) of IVC. Detectable immunoreactivity can be observed both at the perinuclear level and in the emergence of cytoplasmic prolongation (arrows). Bar = 25 µm. 15 = 15,000. HD = hanging drops; LM = liquid marble. Five replicates from all the groups of HD and LM were analysed.

**Figure 6:**
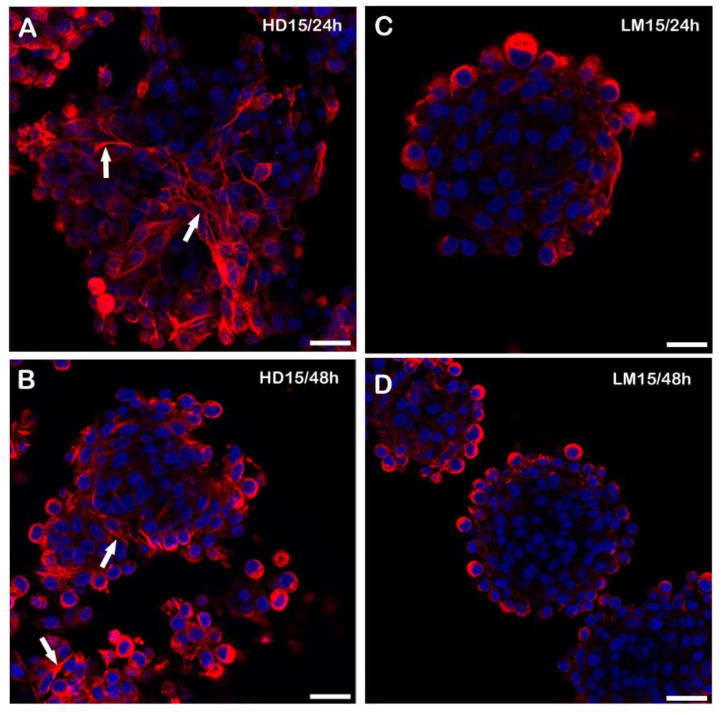
Representative images of immunofluorescence for Ac-T of spheroids at the 15,000-cell concentration. In the left column, from the top to bottom, confocal microscopic observations of HD spheroids at 15,000/24–48 h (**A**,**B**). In the right column, confocal examination of LM spheroids at 15,000/24–48 h (**C**,**D**). Numerous cytoplasmic extensions are detectable (arrows), especially in the peripheral region, where the cells are less attached to each other in the HD spheroids. LM spheroids appear with more compact spheroidal structures with variable sizes, and cytoplasmic processes are mostly absent or barely visible. Bars = 30 µm. Five replicates from all the groups of HD and LM were analysed.

**Figure 7:**
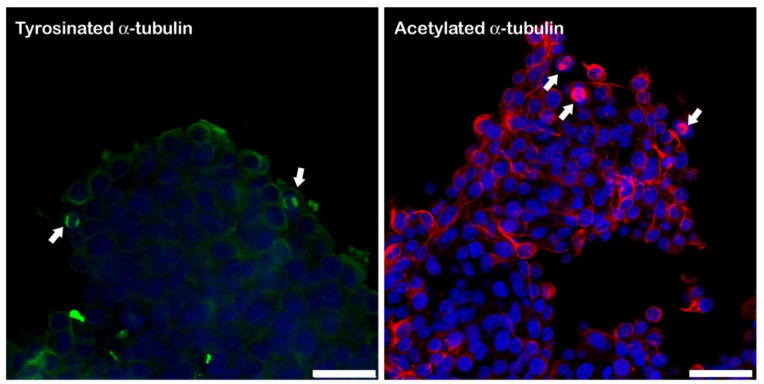
Tyr-T and Ac-T immunofluorescence staining displayed a detectable immunoreactivity for Tyr-T and Ac-T in the mitotic spindles of dividing cells (arrows). Bar = 25 μm.

Tyr-T and Ac-T labelling showed that in both culture systems (LM and HD), immunoreactivity did not show differences in the intensity or distribution throughout the spheroid, even if it did not have evident immunoreactivity in the inner part of the cellular aggregate. Regardless of the cell concentration or length of IVC, Tyr-T was especially evident around the nucleus and in the emergence of cellular processes, particularly at the periphery of the spheroids. Ac-T was evidently distributed along the cellular process; this pattern was more evident in HD spheroids at 24 and 48 h, compared to LM spheroids, whose compactness did not allow any observation of cytoplasmic extensions (Figure 5 and Figure 6). Immunostaining with antibodies against tyrosinated and acetylated tubulin revealed an appreciable immunoreactivity in the mitotic spindles of the spheroid cells, which was particularly noticeable at the periphery of the 3D system (Figure 7).

### 3.3. Western Blot (WB)

**Figure 8:**
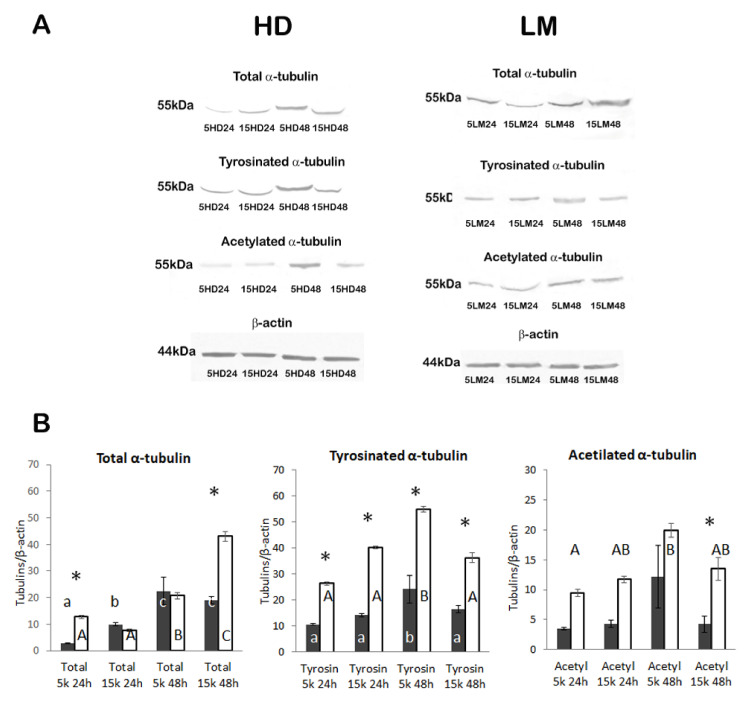
(**A**) Western blot analysis of tubulin PTMs on HD and LM spheroids. Equal protein loading was ascertained using a β-actin antibody. (**B**) Graphs representing the quantification of the optical density of the bands after normalization against β-actin (mean ± standard deviation (SD); *n* = 20, for spheroids at 24 h IVC, and *n* = 3–6 for spheroids at 48 h IVC). Each column is the expression of the mean of three analysed blots. Dark and white columns represent HD and LM spheroids, respectively. Differences among groups were assessed by the ANOVA general linear model followed by Tukey’s post hoc comparisons. Different lowercase letters indicate significant differences between groups within the HD set, and uppercase letters within the LM set: a vs. b = *p* < 0.05; a vs. c = *p* < 0.05; b vs. c = *p* < 0.05; A vs. B = *p* < 0.05; A vs. C = *p* < 0.05; B vs. C = *p* < 0.05. Asterisks (*) indicate a difference between LM and HD at each time point and cell quantity (*p* < 0.05). 15 k = 15,000 cells; 5 k = 5000 cells. HD= hanging drop; LM = liquid marble.

Quantification of the three tubulin PTMs (total alpha, tyrosinated, and acetylated) was performed by evaluation of the optical density of the WB bands with the ImageJ software on three blots for each tubulin. Both the 3D system (HD or LM) and time of IVC (24 or 48 h) significantly affected the levels of total alpha tubulin in the entire set of samples (*p* = 0.01 and *p* < 0.001, respectively) while the cell concentration was not significant (*p* = 0.086). A significant difference in the optical density between the HD and LM spheroids was observed at 24 h with 5000 cells and at 48 h with 15,000 cells (*p* < 0.001 in both cases). Differences between groups within the HD set and within the LM set are shown in the first graph of Figure 8B. In parallel, both the 3D system (HD or LM) and time of IVC (24 or 48 h) significantly affected the levels of tyrosinated tubulin in the entire set of samples (*p* < 0.001 and *p* = 0.001, respectively) while the cell concentration was not significant (*p* = 0.291). A significant difference in the optical density (*p* < 0.001) was observed between HD and LM spheroids at both time points (24 and 48 h) and cell concentrations (5000 and 15,000). Differences between groups within the HD set and within the LM set are shown in the second graph of Figure 8B. Quantification of the acetylated tubulin again showed that both the 3D system (HD or LM) and time of IVC (24 or 48 h) significantly affected the levels of acetylated tubulin in the entire set of samples (*p* < 0.001 and *p* = 0.001, respectively) while the cell concentration approached significance (*p* = 0.059). Nevertheless, a significant difference in the optical density between HD and LM spheroids was observed only at 48 h with 15,000 cells (*p* < 0. 035). No difference was observed between groups within the HD set while differences within the LM set are shown in the third graph of Figure 8B.

## 4. Discussion

The results obtained in the present study demonstrate that fumed silica micro-bioreactors are a robust and cost-effective method for the generation of C6 rat glioma spheroids in terms of morphological changes, 3D cell rearrangements, and the acquisition and maintenance of high plasticity. These observations extend previous evidence indicating that the 3D microenvironment may have a profound influence on cell phenotype and plasticity [45,46]. A simple and reproducible method for generating multicellular clustering is a prerequisite for spheroid-based applications. The general criteria for selecting the most suitable method include production efficiency, spheroid size uniformity, possible damage or influence on cellular physiology, convenience, and suitability for subsequent applications. Cell aggregation is due to mechanical forces that are generated within the different systems and varies between methods.

The HD technique was initially developed to culture stem cell embryoid bodies and has been extensively used for 3D culture conditions due to its simplicity. In fact, to prepare a HD culture, cell suspension drops (30 µL) are deposited onto the underside of the lid of a tissue culture dish. When the lid is inverted, drops are held in place by surface tension and this creates a microgravity environment in each drop that concentrates the cells, which then form single spheroids at the free liquid–air interface. We observed that in the first 24 h of IVC in this system, RGCs tend to settle in the conical region of the semi-rigid surface, producing cytoplasmic extensions, and the possible limited force of gravity forms loose-aggregated clusters (Figure 1). This method allows large production of spheroids and easy control of their size. However, it is labour intensive due to its multistep process, and there is a risk of cell damage in case of media evaporation, requiring constant monitoring of the culture medium.

The LM culture system described in this study provides a non-adherent liquid surface at the bottom that integrates the merits of the spinning and stationary methods, which possibly induce rapid cell aggregation. Moreover, the concave bottom, spherical shape, and internal water flow of each liquid marble allow cells to settle onto the bottom of the liquid marble. In the LM system, on the other hand, the spherical shape makes gravity-driven cell aggregation more intense. Furthermore, in the sphere, small vibrational movements intensify the gravity. Different advantages have been discovered as the reported method does not require specialized equipment or training. Other advantages include the low cost, self-repairing ability of the LM coating, low cell culture medium consumption, high reproducibility, and high gas permeability. Furthermore, the silica nanoparticles are biocompatible, very chemically stable, and due to their transparency, permit constant cell monitoring. The scaffold-free approach for 3D cell culture compartmentalization using LM can serve as a suitable screening platform for compounds and only requires a small volume of liquid.

Encapsulation in super-hydrophobic microbioreactors has previously been shown to support the growth of stem cells [34,37] and lung cancer stem cells [47]. Liu and collaborators [47] compared monolayer cells and spheroids generated in multi-well plates and cultured in LM and observed that the 3D nano-environment promotes in vitro tumour spheroid formation from single or multiple cancer stem cells. Compared to monolayer cells, the spheroids that formed in the LM were more tightly packed and more sensitive to hypoxia conditions, which indicates better gas exchange and an enhanced viability under the treatment of chemotherapeutic drugs and small interfering RNA. In our previous study, ultrastructural analysis of pluripotent cells derived from epigenetically erased fibroblasts suggested that the use of the a liquid marble micro-bioreactor not only encourages cell aggregation, but also boosts the formation and stable maintenance of morphological properties previously described in pluripotent cells derived by [48]. It is well known that culture parameters, including the cell type, seeding density, medium composition, and length of incubation, can influence cell aggregation and spheroid formation. These parameters are determinants of the cell aggregation efficiency and the uniformity of the spheroid size and shape. Our results showed how both parameters (seeding density and time of incubation) influenced cell clustering. Indeed, in both systems, larger spheroids were observed when the seeding density or time of incubation was increased and in the LM compared to the HD system. Previous studies using cancer lung cells showed how cells cultured in an LM microbioreactor had different levels of e-cadherin expression compared to those cultured in a monolayer [47]. The different methods used to achieve the growth and compaction of spheroids certainly influence the microtubular composition pattern of the glioblastoma cells. The confocal analysis of the spheroids generated with the LM system showed an actual sphere because of the flat-bottom layer present during the culture in non-adherent culture plastic dishes. Cells inside the spheroid showed a compact configuration and maintained a constant phenotype. Moreover, cells localized in the external section of the spheroids showed a different grade of mitotic cell replication, which indicates the high viability of the glioma cells cultured in this system. Fluorescence confocal microscope observation showed good immunoreactivity for the two tubulin PTMs examined in this work (Tyr-T and Ac-T). These results confirm a previous work, where it was observed, for the first time, that Tyr-T and Ac-T are the most abundant tubulin modifications in RGC in a 2D system [40,43]. Moreover, the distribution of the two tubulin PTMs was clearly visible in the 3D cultured cells. Tyr-T was most appreciable around the nucleus and in the emergence of the cell process while Ac-T was detectable along the cell extensions. The most interesting data on tubulin PTMs in the present work, however, relate to the Western blot and the quantification of the bands. For the first time, in fact, the behaviour and quantification of PTMs of these tubulins in C6 RGCs grown in systems, such as HD and LM spheroids, were investigated. The data derived from WB showed a certain dynamism expressed by the increased levels of Tyr-T and also the cytoskeletal stability displayed by the level of Ac-T. It is well known that the microtubular physiology is regulated by the presence of tubulin PTMs, which make microtubules more dynamic or more stable. Indeed, the same microtubule can be “mixed” along the protofilament, with an alternation of traits rich in Tyr-T, a widely considered marker of microtubular dynamism [49], and traits with greater abundance of Ac-T, considered a marker of stable microtubules [50]. Depending on the biological structure, these two tubulin PTMs can prevail over each other. Here, we observed that during the growth of the spheroids in both LM and HD systems, an increase in the Tyr-T could be appreciated. Usually, this behaviour is appreciable in those biological structures where rapid adaptation and continuous elongation of the microtubules are required [51]; it is typical of continuously evolving structures, as these spheroids seem to be capable of growing and changing their spatial disposition over time. As the hours pass, and with the increase in the size of the spheroids, we detected a significant increase in Ac-T, which indicates the tendency of the organoid to stabilize and consolidate its spherical structure. Indeed, it was reported that Ac-T increases in curved structures subjected to bending, giving them greater resistance [52,53].

The use of LM microbioreactors allows the scaling down of experiments and is therefore amenable for high-throughput applications and to study the effect of paracrine/autocrine signalling of the rich environment established within the microbioreactor.

## 5. Conclusions

In conclusion, by comparing the morphology and microtubular architecture of spheroids from C6 rat glioma cells developed by the LM or HD methodology, our findings demonstrate that the use of the fumed silica microbioreactor boosts the induction and maintenance of a high plasticity state in glioma cells. The proposed in vitro 3D culture system provides an appropriate in vitro culture technique for studying glioblastoma, inducing distinctive 3D cell rearrangement and specific cell–cell interactions. Considering that the establishment of appropriate 3D in vitro culture systems for studying cancer and other biological processes is of general importance, we believe that the fumed silica microbioreactors may represent a notable tool for advancing knowledge on glioblastoma. The expression levels of tubulin PTMs under these conditions can be used to evaluate the efficacy of new anticancer therapies. The transparent and gas-permeable LM will be valuable in a wide range of research fields, where spheroid cultures, in situ monitoring, and high-throughput drug sensitivity tests are needed.

## Data Availability

The data produced during the current study are available from the corresponding author on reasonable request.

## Supplementary Materials

The following are available online at https://www.mdpi.com/article/10.3390/biology11040492/s1 containing images of the original blots added in the revised version of the manuscript. Table S1: Clustering during IVC of C6 RGC with HD or LM techniques.
