# Supplementary material for: Microtubular Assessment of C6 Rat Glioma Cell Spheroids Developed in Transparent Liquid Marbles or Hanging Drops
Source: Biology (Basel). 2022 Mar 23;11(4):492. doi: 10.3390/biology11040492 (PMC9031767; doi:10.3390/biology11040492)
Supplement: Supplementary file 1 [file biology-11-00492-s001.zip › biology-1611346-supplementary.pdf]

Table S1. Clustering during IVC of C6 RGC with HD or LM techniques. The data represent the coefficients of variation (CV) of twenty spheroids (n=20) per experimental group (Gr) at 24h and 48h of IVC. 5K=5,000 cells; 15K=15,000 cells. HD=Hanging Drops; LM=Liquid Marble.

	**HD**				**LM**			
**Gr**	5K/24h	15K/24h	5K/48h	15K/48h	5K/24h	15K/24h	5K 48h	15K/48h
**CV**	50,00	40,54	48,05	8,81	31,58	34,62	24,07	15,77
