# Supplementary figures and images for: Microtubular Assessment of C6 Rat Glioma Cell Spheroids Developed in Transparent Liquid Marbles or Hanging Drops
Source: Biology (Basel). 2022 Mar 23;11(4):492. doi: 10.3390/biology11040492 (PMC9031767; doi:10.3390/biology11040492)

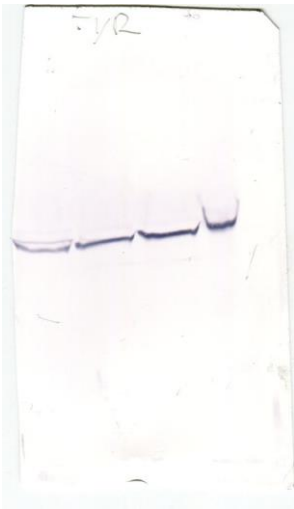

LM spheroids

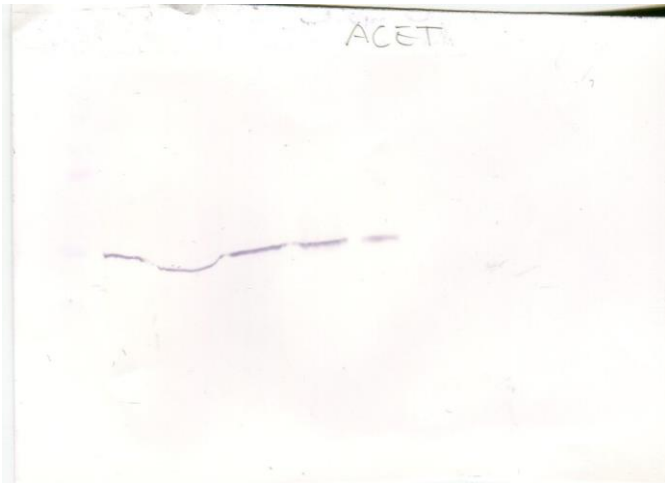

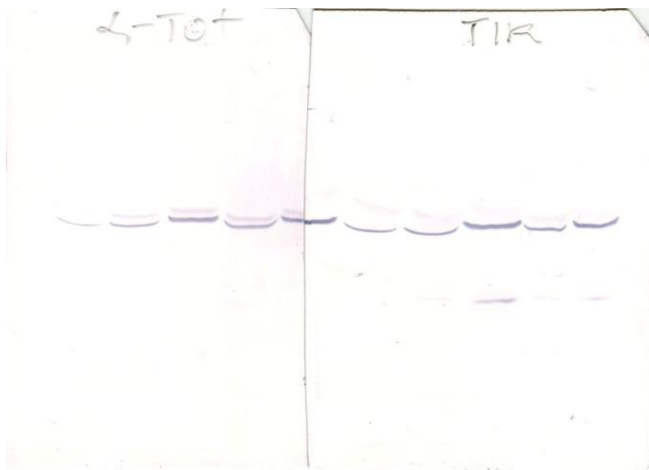

HD spheroids

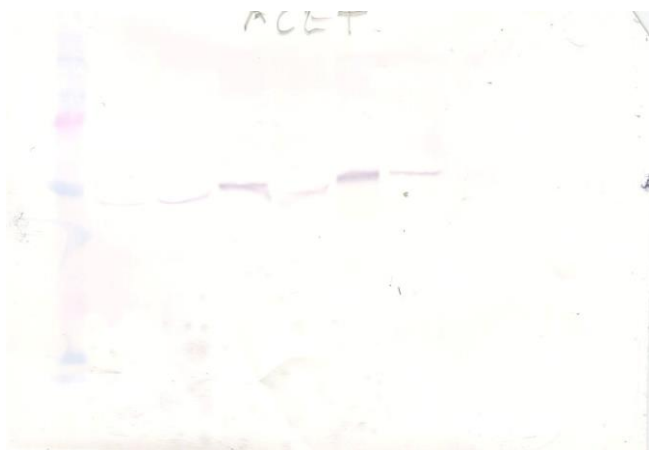

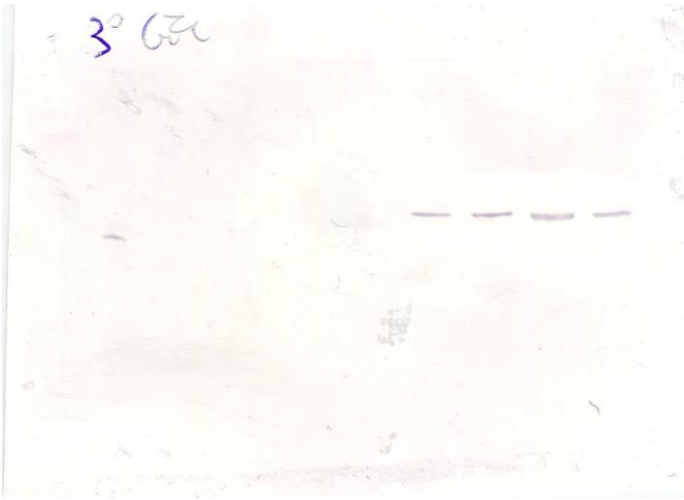

Tyrosinated LM spheroids

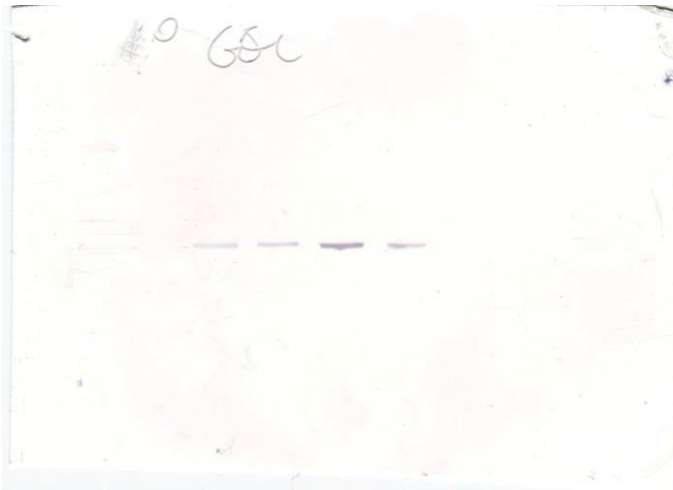

Acetylated HD spheroids

Supplement: Supplementary file 1 [file biology-11-00492-s001.zip › biology-1611346-original image.pdf]
